# Efficacy and safety of once weekly selinexor 40 mg versus 60 mg with pomalidomide and dexamethasone in relapsed and/or refractory multiple myeloma

**DOI:** 10.3389/fonc.2024.1352281

**Published:** 2024-05-17

**Authors:** Darrell White, Gary J. Schiller, Sumit Madan, Suzanne Lentzsch, Evgeni Chubar, Noa Lavi, Dane R. Van Domelen, Ohad S. Bentur, Muhamed Baljevic

**Affiliations:** ^1^ Department of Medicine, Dalhousie University and Queen Elizabeth II Health Sciences Centre, Halifax, NS, Canada; ^2^ Department of Medicine, David Geffen School of Medicine at University of California, Los Angeles (UCLA), Los Angeles, CA, United States; ^3^ Department of Medicine, Banner MD Anderson Cancer Center at Banner University Medical Center, Phoenix, AZ, United States; ^4^ Department of Medicine, Columbia University, New York, NY, United States; ^5^ Clalit Health Services, Ha’Emek Medical Center, Afula, Israel; ^6^ Department of Hematology and Bone Marrow Transplantation, Haifa, Israel; ^7^ Karyopharm Therapeutics Inc., Newton, MA, United States; ^8^ Department of Medicine, Vanderbilt University Medical Center, Nashville, TN, United States

**Keywords:** selinexor, once weekly dose, optimal triplet combination, relapsed/refractory multiple myeloma, pomalidomide

## Abstract

**Objective:**

To identify the optimal dose of selinexor in combination with pomalidomide and dexamethasone (SPd).

**Methods:**

An analysis of efficacy and safety of 2 once-weekly selinexor regimens (60 mg and 40 mg) with pomalidomide and dexamethasone (SPd-60 and SPd-40, respectively) given to patients with relapsed/refractory multiple myeloma (RRMM) in the STOMP (NCT02343042) and XPORT-MM-028 (NCT04414475) trials.

**Results:**

Twenty-eight patients (60.7% males, median age 67.5 years) and 20 patients (35.0% males, median age 65.5 years) were analyzed in the SPd-40 and SPd-60 cohorts, respectively. Overall response rate was 50% (95% confidence interval [CI] 30.6-69.4%) and 65% (95% CI 40.8-84.6%), respectively. Very good partial response or better was reported in 28.6% (95% CI 13.2-48.7%) and 30.0% (95% CI 11.9-54.3%) of patients, respectively. Among 27 responders in both cohorts, the 12-month sustained response rate was 83.3% (95% CI 64.7-100.0%) for SPd-40 and 28.1% (95% CI 8.9-88.8%) for SPd-60. Median progression-free survival was 18.4 months (95% CI 6.5 months, not evaluable [NE]) and 9.5 months (95% CI 7.6 months-NE) for SPd-40 and SPd-60, respectively. Twenty-four-month survival rates were 64.2% (95% CI 47.7-86.3%) for SPd-40 and 51.1% (95% CI 29.9-87.5%) for SPd-60. Treatment-emergent adverse events (TEAEs) included neutropenia (all grades: SPd-40 64.3% versus SPd-60 75.0%), anemia (46.4% versus 65.0%), thrombocytopenia (42.9% versus 45.0%), fatigue (46.4% versus 75.0%), nausea (32.1% versus 70.0%) and diarrhea (28.6% versus 35.0%).

**Conclusion:**

The all-oral combination of SPd exhibited preliminary signs of efficacy and was generally tolerable in patients with RRMM. The overall risk-benefit profile favored the SPd-40 regimen.

## Highlights

- Despite advancements in treatment, multiple myeloma (MM) remains without a cure and nearly always develops refractoriness to the 3 primary classes of standard therapies (immunomodulatory drugs, proteasome inhibitors and anti-CD38 monoclonal antibodies (αCD38 mAbs). Post-treatment with αCD38 mAbs, no standard of care has been established, and data are limited on the effectiveness of therapeutic triplets based on pomalidomide and low-dose dexamethasone (Pd) in this setting. Selinexor is an oral selective inhibitor of exportin 1-mediated nuclear export. To identify the optimal dose of combined treatment with selinexor, pomalidomide and dexamethasone, we have analyzed the efficacy and safety of two selinexor regimens (60 mg and 40 mg) with pomalidomide and dexamethasone (SPd-60 and SPd-40, respectively) administered once-weekly to patients with relapsed/refractory multiple myeloma in the STOMP (NCT02343042) and XPORT-MM-028 (NCT04414475) trials. Overall, the all-oral combination of weekly SPd was generally tolerable and suggests that the combination is effective in this patient population. Overall response rate was greater in the SPd-60 cohort, but both progression-free survival and treatment duration were longer in the SPd-40 cohort despite a higher rate of triple-class refractory disease at baseline. The SPd-40 cohort had a better AE profile than the SPd-60 group, suggesting a better benefit-risk profile compared to SPd-60.

## Introduction

Despite advancements in treatment, multiple myeloma (MM) remains without a cure, nearly always developing refractoriness to 3 primary classes of standard anti-MM therapies: immunomodulatory drugs (IMiDs), proteasome inhibitors (PIs) and anti-CD38 monoclonal antibodies (αCD38 mAbs).

Selinexor (XPOVIO, Karyopharm Therapeutics Inc, Newton, MA, USA), an oral selective inhibitor of exportin 1-mediated nuclear export, was first approved by the United States Food and Drug Administration (FDA) after the STORM clinical trial. In that study, patients with heavily pretreated MM received oral selinexor 80 mg twice a week (BIW) together with dexamethasone (Sd) (1). Although this dose showed a response in treated patients, adverse events (AEs) included grade 3 or higher thrombocytopenia, neutropenia, anemia, fatigue, nausea and hyponatremia. Eighteen percent of patients discontinued treatment due to AEs related to treatment.

BOSTON (NCT03110562) was a phase 3 randomized trial that compared the safety and efficacy of a once-weekly regimen of oral selinexor (100 mg) together with subcutaneous bortezomib and low-dose dexamethasone (SVd) to those of the standard regimen comprising twice-weekly bortezomib in combination with low-dose dexamethasone (Vd). The results of the study showed that patients treated with SVd had significantly higher overall response rate (ORR), a significantly higher rate of very good partial response (VGPR) or better responses, and significantly longer progression-free survival (PFS), compared to patients treated with Vd. Lower rates of grade 2 or higher peripheral neuropathy were also observed in the SVd cohort compared to the Vd cohort (2). In the same study, selinexor dose reductions due to AEs from the 100 mg starting dose led to decreased AE rates and were related to improved efficacy (3). Following the study, the SVd combination was approved for the treatment of adult patients with MM who were previously treated with 1 or more therapies (4).

The ongoing phase 1b/2 Selinexor and Backbone Treatments of Myeloma Patients (STOMP) trial (NCT02343042) is a multi-center, multi-arm, open-label study in which varied dosing of selinexor is being evaluated in several triplet and quadruplet combinations in patients with newly diagnosed and relapsing/refractory multiple myeloma (RRMM) (5–7). One of the triplet combinations being evaluated in this study is selinexor, pomalidomide and dexamethasone (SPd).

There are limited data on the effectiveness of pomalidomide and low-dose dexamethasone (Pd)-based triplets in the evolving post-anti CD38 mAb treatment landscape, in which there is no standard of care (8, 9). Prior studies that have evaluated the efficacy of Pd in patients with MM refractory to both lenalidomide and bortezomib, reported an ORR of 28% and a median PFS of 3.7 months (10). In phase 1/2 of the STOMP trial, the efficacy of the 28-day treatment cycle SPd combination was assessed at selinexor doses of 60 mg to 80 mg BIW (weeks 1 to 3 only) or 40 mg to 100 mg once weekly (QW) in combination with pomalidomide 2 mg to 4 mg once daily (QD, days 1 to 21) and dexamethasone 40 mg weekly. In phase 2 of the study, in line with the shift away from the maximum tolerated dose and evolving dose optimization paradigms in clinical development (11), two QW selinexor regimens with pomalidomide 4 mg QD were tested: 60 mg (SPd-60) and 40 mg (SPd-40). The XPORT-MM-028 study (NCT04414475) is a parallel ongoing phase 2b trial with similar objectives and eligibility criteria to those of the STOMP trial, evaluating selinexor in different combinations and doses, including SPd-40, in patients with RRMM.

The aim of the analysis described in this paper was to identify the optimal dose of SPd by comparing the safety and efficacy of SPd-60 (from STOMP Phase 1/2) and SPd-40 (from STOMP Phase 2 and XPORT-MM-028).

## Methods

The study protocols for STOMP (See https://clinicaltrials.gov/study/NCT02343042 for details about the trial) and XPORT-MM-028 (https://clinicaltrials.gov/study/NCT04414475) received approval from the institutional review board or the independent ethics committee at each participating center. These studies were conducted in compliance with the Declaration of Helsinki, the International Council on Harmonization-Good Clinical Practice, and local laws. Before enrollment, all patients provided written informed consent.

### Patients

The SPd arm of the STOMP study enrolled patients with relapsed and/or refractory MM who had ≥2 prior cycles of lenalidomide and a PI (in combination or in distinct therapeutic regimens [not for maintenance]), an Eastern Cooperative Oncology Group (ECOG) performance status of 0 to 2, adequate renal function defined as creatinine clearance ≥45 mL/min, hematopoietic function (total white blood cell count ≥1500/mm^3^, absolute neutrophil count ≥1,000/mm^3^, hemoglobin ≥8 g/dL, platelet count ≥75,000/mm^3^ [protocol versions 2-6], or ≥30,000/mm^3^ for those in whom 50% or more of bone marrow nucleated cells were plasma cells [protocol version 2], platelet count ≥150,000/mm^3^ [protocol versions 7-9], or platelet count ≥100,000/mm^3^ [protocol versions 10-11]; for expansion cohorts, platelet counts ≥50,000/mm^3^; platelets ≥30,000/mm^3^ were acceptable in expansion cohorts for those in whom 50% or more of bone marrow nucleated cells were plasma cells), cardiac function (left ventricular ejection fraction ≥50%), and adequate hepatic function (total bilirubin < 2x upper limit of normal [ULN] and both aspartate aminotransferase and alanine aminotransferase < 2.5x ULN). It was required that patients had measurable disease (serum M-protein ≥0.5 g/dL or for quantitative immunoglobulin A ≥0.5 g/dL, urine M-protein ≥200 mg/day, or involved serum free light chain ≥100 mg/L). In the expansion arm, patients were not included if they had disease refractory to pomalidomide (protocol versions 5-11). Other key eligibility criteria have been previously outlined (5–7).

Eligibility criteria for the XPORT-MM-028 study were similar, but in contrast to the SPd arm of the STOMP study, XPORT-MM-028 excluded patients previously treated with pomalidomide.

### Outcomes

Disease response was assessed in study-eligible patients according to the response criteria of the International Myeloma Working Group (12). The definition of ORR was the proportion of patients who achieved a confirmed partial response (PR) or better. Clinical benefit rate (CBR) was defined as the proportion of patients who achieved a confirmed minimal response (MR) or better. PFS was defined as the duration from the first dose of study treatment to the first confirmation of progressive disease (PD) or death. If patients discontinued treatment before confirmation of PD or death, or if they were still receiving the study treatment at the time of the analysis and had no confirmed PD, DOR and PFS were censored at the latest response assessment, before, or on, the date of treatment discontinuation (where applicable). OS was defined as the duration from the first dose of study treatment to death from any cause. Time to response (TTR) was defined for responders as the duration from the first dose of study treatment to the first confirmation of PR or better. DOR was defined for responders as the duration from the first confirmation of PR or better to the first confirmation of PD or death.

Patients were categorized as having refractory MM to a specific drug if their prior treatment with the drug led to a best overall response (BOR) of stable disease (SD) or PD, or if their disease progressed either during treatment or within 60 days following the completion of treatment, as per the IMWG Consensus Guidelines on Uniform Reporting in Clinical Trials (13).

Conversely, patients were not classified as having refractory disease if they met these criteria in one treatment regimen but were subsequently re-treated with the same drug in a later regimen, achieving a PR or better without experiencing PD during treatment or within 60 days after treatment cessation.

The investigator evaluated treatment-emergent adverse events (TEAEs) according to the National Cancer Institute Common Terminology Criteria for Adverse Events v4.03.

### Statistical design

Each expansion cohort in STOMP (SPd-40 and SPd-60) was designed to test the null hypothesis that ORR ≤30% against a one-sided alternative. The following two-stage design provided approximately 80% power at a one-sided α=0.1 assuming a true ORR of 55%: The null hypothesis was rejected if there were at least 4 responders among the first 10 patients enrolled (otherwise stop for futility) and subsequently, if there were at least 9 responders out of a total of 20 enrolled patients.

The SPd-40 arm of XPORT-MM-028 was designed to test the same hypothesis. The following single-stage design provided approximately 80% power at a one-sided α=0.1 assuming a true ORR of 60%: the null hypothesis was rejected if there were at least 7 responders among 14 patients enrolled.

While the statistical designs focused on single-arm testing of ORR, the purpose of testing two different selinexor doses in across two Phase 2 studies was to compare their overall safety and efficacy profiles and determine which regimen, if any, would be tested in Phase 3. The *ad hoc* nature of the analysis serves as guidance for dosing in future clinical trials testing selinexor combinations.

### Statistical analysis

Summary statistics were calculated separately for each dose. Categorical variables were summarized by frequencies and percentages. Continuous variables were summarized by median and range. Exact two-sided 95% confidence intervals (CI) were calculated for binary efficacy endpoints (ORR, CBR, and VGPR or better rate), along with odds ratio with 95% CI comparing SPd-40 to SPd-60 based on Cochran-Mantel-Haenszel test. Time-to-event endpoints were summarized by Kaplan-Meier methodology, along with hazard ratio (HR) with 95% CI comparing SPd-40 to SPd-60 based on Cox proportional hazards model with Efron’s method of handling ties. Statistical analyses were done using SAS version 9.4 (SAS Institute, Cary, NC).

## Results

### Baseline demographic and clinical characteristics

At the data cutoff date (June 30, 2023), 28 study-eligible patients (60.7% males) with a median age of 67.5 years (range 48-79) were enrolled in the SPd-40 cohort and 20 patients (35.0% males) with a median age of 65.5 years (range 37-85) were enrolled in the SPd-60 cohort (**Table 1**). No further enrollment is planned in any cohort. The median number of prior therapy lines was 2 in both cohorts. The percentage of patients with ECOG performance status 1-2 was lower in the SPd-40 compared to the SPd-60 cohort (71.4% versus 90.0%).

**Table 1:** Patient demographics and clinical characteristics.

Characteristic	SPd-40 N = 28	SPd-60 N = 20
**Age, years, median (range)**	67.5 (48–79)	65.5 (37–85)
Sex, n (%)		
Male	17 (60.7)	7 (35.0)
Female	11 (39.3)	13 (65.0)
**Duration from initial diagnosis to first dose of study treatment, years, median (range)**	4.3 (0.8-25.0)	3.4 (1.1-9.2)
ISS stage at initial diagnosis, n (%)		
I	7 (25.0)	7 (35.0)
II	6 (21.4)	3 (15.0)
III	8 (28.6)	3 (15.0)
Unknown	7 (25.0)	7 (35.0)
Baseline ECOG performance status, n (%)		
0	8 (28.6)	2 (10.0)
1	16 (57.1)	14 (70.0)
2	4 (14.3)	4 (20.0)
**Genetic abnormalities at initial diagnosis, n (%)**	11 (39.3)	8 (40.0)
del(17p)	0	1 (12.5)
t(4,14)	3 (27.3)	1 (12.5)
t(14,16)	1 (9.1)	1 (12.5)
Any of del(17p), t(4,14), or t(14,16), n (%)	4 (36.4)	3 (37.5)
**Number of prior lines of therapy, median (range)**	2.0 (1–5)	2.0 (1–9)
Number of prior lines of therapy, n (%)		
1	3 (10.7)	3 (15.0)
2	12 (42.9)	8 (40.0)
3	5 (17.9)	4 (20.0)
4	4 (14.3)	2 (10.0)
≥5	4 (14.3)	3 (15.0)
Previously exposed; refractory, n (%)		
Bortezomib	26 (92.9); 21 (75.0)	17 (85.0); 8 (40.0)
Carfilzomib	10 (35.7); 10 (35.7)	12 (60.0); 9 (45.0)
Ixazomib	7 (25.0); 7 (25.0)	5 (25.0); 3 (15.0)
Thalidomide	5 (17.9); 3 (10.7)	1 (5.0); 0
Lenalidomide	28 (100.0); 21 (75.0)	20 (100.0); 17 (85.0)
Pomalidomide	1 (3.6); 0	4 (20.0); 3 (15.0)
Daratumumab	16 (57.1); 16 (57.1)	6 (30.0); 4 (20.0)
Isatuximab	0; 0	0; 0
Elotuzumab	0; 0	1 (5.0); 0
Belantanab mafodotin	1 (3.6); 1 (3.6)	0; 0
PI (bortezomib or carfilzomib or ixazomib)	28 (100.0); 26 (92.9)	20 (100.0); 15 (75.0)
IMiD (thalidomide or lenalidomide or pomalidomide)	28 (100.0); 21 (75.0)	20 (100.0); 17 (85.0)
αCD38 mAb (daratumumab or isatuximab)	16 (57.1); 16 (57.1)	6 (30.0); 4 (20.0)
**Triple-class exposed; refractory**	16 (57.1); 12 (42.9)	6 (30.0); 4 (20.0)
**Penta-exposed; refractory**	1 (3.6); 0	3 (15.0); 1 (5.0)
**Autologous stem cell transplant, n (%)**	20 (71.4)	16 (80.0)

αCD38, anti-CD38; ECOG, Eastern Cooperative Oncology Group; IMiD, immunomodulatory agent; ISS, International Staging System for multiple myeloma; mAb, monoclonal antibody; PI, proteasome inhibitor; SPd-40, 40 mg selinexor once weekly + 4 mg pomalidomide once daily + dexamethasone; SPd-60, 60 mg selinexor once weekly + 4 mg pomalidomide once daily + dexamethasone.

Among patients treated with SPd-40 and SPd-60, 92.9% and 75.0%, respectively, had MM refractory to a PI, 75.0% and 85.0%, respectively, had disease refractory to an IMiD, and 57.1% and 20.0%, respectively, had MM refractory to an αCD38 mAb. The percentage of patients with triple-class refractory MM was 42.9% and 20.0% in the SPd-40 and SPd-60 cohorts, respectively. Previous exposure to pomalidomide was reported in 1 patient (3.6%) in the SPd-40 cohort and 4 patients (20.0%) in the SPd-60 cohort. No patient in the SPd-40 cohort had MM refractory to pomalidomide compared to 3 patients (15%) in the SPd-60 cohort. One pomalidomide-refractory patient treated with SPd-60 had been enrolled in the dose-expansion phase of the study; the two other patients refractory to pomalidomide were erroneously enrolled. All pomalidomide-refractory patients were included in the efficacy and safety analyses. Most patients had autologous stem cell transplantation (71.4% and 80.0% in the SPd-40 and SPd-60 cohorts, respectively).

### Efficacy of treatment

The efficacy analysis is based on the pooled dosing cohorts across two clinical trials; as such, the results reported herein are meant to be interpreted as primarily descriptive in nature only.

At the time of data cutoff, 5 patients in the SPd cohort were still being treated. All of them were responders with more than 23 months of therapy. Four additional patients stopped treatment with SPd and were in survival follow-up (3 of them were in the SPd-40 cohort). The median duration of exposure in the SPd-40 and SPd-60 cohorts was 28 and 22 weeks, respectively, the median average weekly selinexor dose was 37.7 mg and 46.6 mg, respectively, and the median relative selinexor dose intensity was 94.3% and 77.6%, respectively (**Table 2**). ORR was 50% (95% CI 30.6%, 69.4%) and 65% (95% CI 40.8%, 84.6%), respectively and CBR was 67.9% (95% CI 47.6%, 84.1%) and 75.0% (95% CI 50.9%, 91.3%), respectively (**Table 3**). The percentage of patients with VGPR or better was 28.6% (95% CI 13.2%, 48.7%) in the SPd-40 cohort and 30.0% (95% CI 11.9%, 54.3%) in the SPd-60 cohort. One patient in the SPd-60 cohort who achieved a response of sCR underwent minimal residual disease testing via next-generation sequencing (clono-SEQ, version 2.0, Adaptive Biotechnologies) and was found to be positive for residual malignant clones in >1/10,000 cells.

**Table 2:** Selinexor exposure.

Characteristic	SPd-40 N=28	SPd-60 N=20
Duration of exposure^a^, weeks, median (range)	28.0 (2, 113)	22.0 (7, 114)
Average weekly selinexor dose^b^, mg/week, median (range)	37.7 (9.3, 45.7)	46.6 (28.3, 60.0)
Relative selinexor dose intensity^c^, %, median (range)	94.3 (23, 114)	77.6 (47, 100)

a Calculated as (date of last dose - date of first dose + 1)/7, rounded up to the nearest integer.

b Calculated as (total dose received)/(duration of exposure).

c Calculated as (average selinexor dose received per week)/(planned selinexor dose per week)*100.

SPd-40 = 40 mg selinexor once weekly + 4 mg pomalidomide once daily + dexamethasone, SPd-60 = 60 mg selinexor once weekly + 4 mg pomalidomide once daily + dexamethasone.

**Table 3:** Efficacy of SPd-40 versus SPd-60.

	SPd-40 N = 28	SPd-60 N = 20
Overall response rate, n (%) [95% CI]	14 (50.0) [30.6, 69.4]	13 (65.0) [40.8, 84.6]
Odds ratio (95% CI)	0.54 (0.17, 1.75)	
Clinical benefit rate, n (%) [95% CI]	19 (67.9) [47.6, 84.1]	15 (75.0) [50.9, 91.3]
Odds ratio (95% CI)	0.70 (0.19, 2.55)	
Very good partial response or better, n (%) [95% CI]	8 (28.6) [13.2, 48.7]	6 (30.0) [11.9, 54.3]
Odds ratio (95% CI)	0.93 (0.26, 3.29)	
Progression-free survival, months, median (95% CI)	18.4 (6.5, NE)	9.5 (7.6, NE)
Hazard ratio (95% CI)	0.68 (0.28, 1.68)	
Time to response, months, median (95% CI)	1.0 (1.0, 6.0)	1.0 (0.9, NE)
Hazard ratio (95% CI)	0.86 (0.39, 1.91)	
Duration of response, months, median (95% CI)	NR (17.5, NE)	8.6 (3.9, NE)
Hazard ratio (95% CI)	0.23 (0.06, 0.80)	
Overall survival, months, median (95% CI)	NR (12.9, NE)	NR (9.3, NE)
Hazard ratio (95% CI)	0.83 (0.31, 2.23)	

CI, confidence interval; NE, not evaluable; NR, not reached; SPd-40, 40 mg selinexor once weekly + 4 mg pomalidomide once daily + dexamethasone; SPd-60, 60 mg selinexor once weekly + 4 mg pomalidomide once daily + dexamethasone.

Among the 27 responders in both cohorts, median TTR was 1.0 month in both cohorts (SPd-40 95% CI: 1.0, 6.0 months; SPd-60 95% CI: 0.9 month, not evaluable [NE]). Median DOR in the SPd-40 cohort was not reached (95% CI 17.5 months, NE) and was 8.6 months in the SPd-60 cohort (95% CI 3.9 months, NE). The 12-month sustained response rate was 83.3% (95% CI 64.7%, 100.0%) for SPd-40 and 28.1% (95% CI 8.9%, 88.8%) for SPd-60.

**Figure 1:**
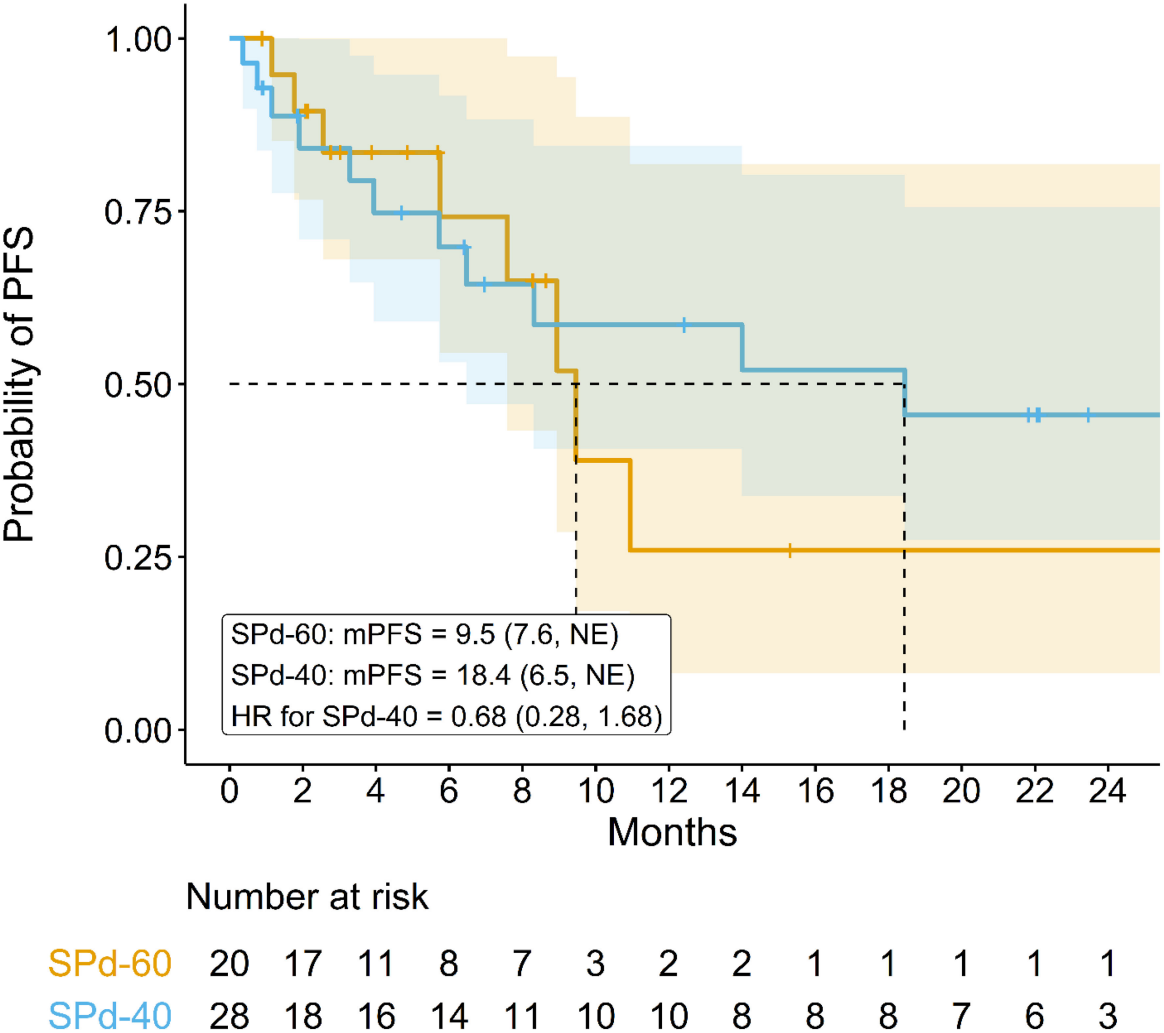
Kaplan-Meier curve comparing progression-free survival in patients who received SPd-40 versus those who received SPd-60.

Median PFS was 18.4 months (95% CI 6.5 months, NE) for SPd-40 and 9.5 months (95% CI 7.6 months, NE) for SPd-60 (**Figure 1**), with an HR of 0.68 (95% CI 0.28, 1.68) favoring SPd-40. Notably, some separation between the PFS curves was observed after approximately 9 months favoring SPd-40; however, as there were only 20 events, considerable statistical uncertainty remains.

**Figure 2:**
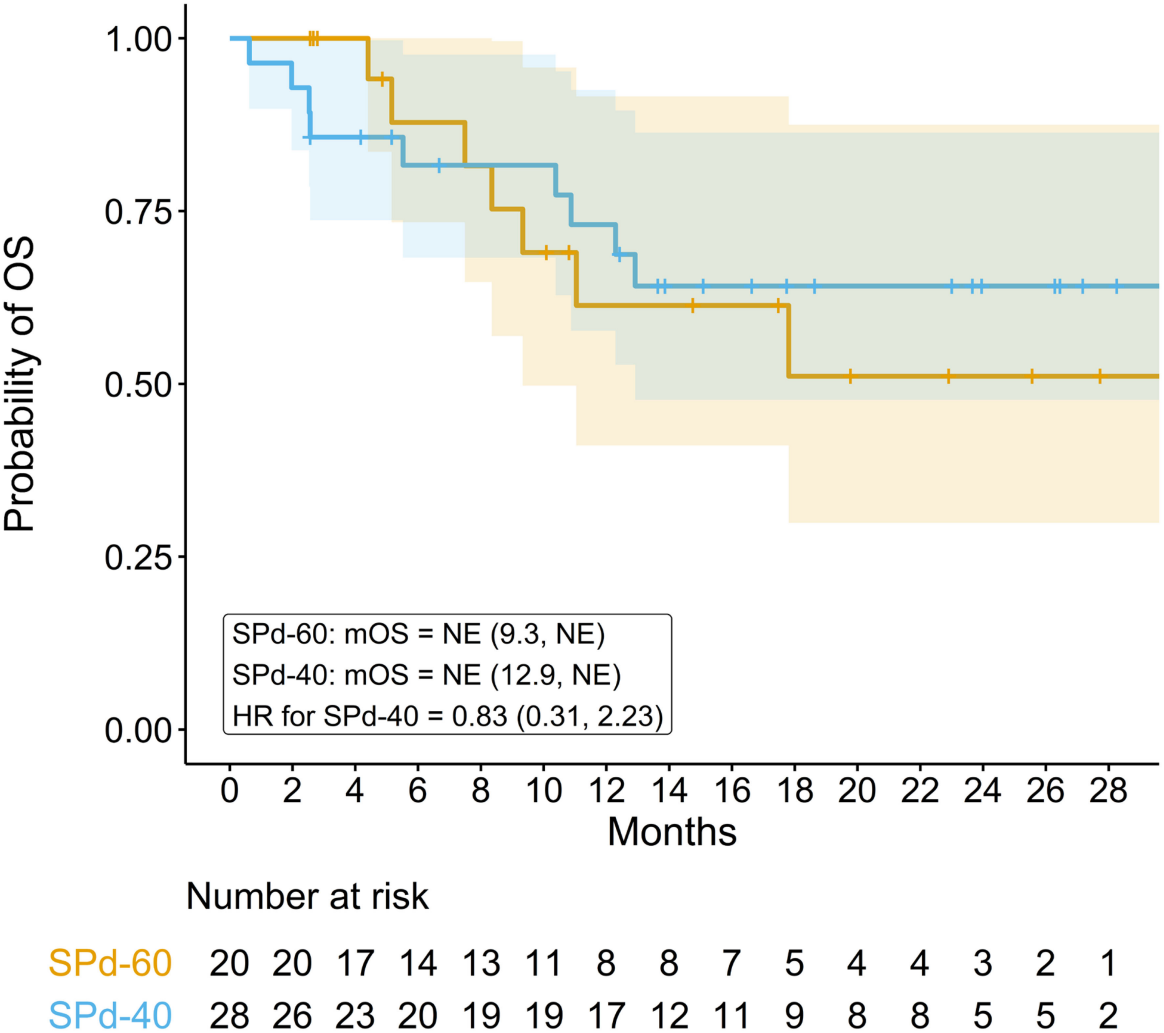
Kaplan-Meier curve comparing overall survival in patients who received SPd-40 versus those who received SPd-60.

Median OS was not reached in either dose cohort after a median follow-up time of 18.6 months for the SPd-40 cohort and 17.5 months for the SPd-60 cohort (95% CI 12.9 months, NE; 9.3 months, NE, respectively, **Figure 2**). Twenty-four-month survival rates were 64.2% (95% CI 47.7%, 86.3%) for SPd-40 and 51.1% (95% CI 29.9%, 87.5%) for SPd-60. The HR was 0.83 (95% CI 0.31, 2.23), slightly favoring SPd-40 but imprecise because it is based on only 16 total events.

### Safety and tolerability

TEAEs were reported in all 48 patients (100%); 25 patients (85.7%) in the SPd-40 cohort and 19 patients (95.0%) in the SPd-60 cohort had maximum grade 3-4 TEAEs. Fifteen patients (53.6%) in SPd-40 and 9 patients (45.0%) in SPd-60 had serious TEAEs.

The most prevalent hematologic TEAEs were neutropenia (all grades: SPd-40 64.3% versus SPd-60 75.0%), with 2 maximum grade 3 and one maximum grade 4 febrile neutropenia in the SPd-40 cohort and one maximum grade 3 febrile neutropenia in the SPd-60 cohort), anemia (46.4% versus 65.0%) and thrombocytopenia (42.9% versus 45.0%). Notably, no high-grade hemorrhages were reported. Non-hematologic TEAEs were mostly reversible and transient, including fatigue (46.4% versus 75.0%), nausea (32.1% versus 70.0%) and diarrhea (28.6% versus 35.0%) (**Table 4**). Fourteen patients (50.0%) in the SPd-40 cohort and 12 patients (60.0%) in the SPd-60 cohort had at least one TEAE of system organ class ‘Infections and infestations’, the most prevalent of which were pneumonia (25.0% versus 15.0%) and COVID-19 (21.4% versus 0%). Only 1 Grade 5 AE - intracranial hemorrhage was reported in one patient in the SPd-40 cohort. This patient, whose BOR was VGPR, discontinued SPd-40 due to PD after 44 weeks of treatment. The patient died 7 days after the end of treatment (14 days after the last treatment dose). This AE was considered by the investigator as unrelated to the study treatment.

**Table 4:** Treatment-emergent adverse events in >25% of patients in either cohort.

	SPd-40 N=28 n (%)		SPd-60 N=20 n (%)	
	All grades	Grade 3-4	All grades	Grade 3-4
Hematological TEAEs				
Neutropenia	18 (64.3)	18 (64.3)	15 (75.0)	12 (60.0)
Anemia	13 (46.4)	6 (21.4)	13 (65.0)	5 (25.0)
Thrombocytopenia	12 (42.9)	7 (25.0)	9 (45.0)	5 (25.0)
Leukopenia	3 (10.7)	1 (3.6)	6 (30.0)	1 (5.0)
Non-hematological TEAEs				
Fatigue	13 (46.4)	1 (3.6)	15 (75.0)	3 (15.0)
Nausea	9 (32.1)	2 (7.1)	14 (70.0)	0
Diarrhea	8 (28.6)	*	7 (35.0)	0
Arthralgia	5 (17.9)	1 (3.6)	7 (35.0)	0
Dizziness	9 (32.1)	0	3 (15.0)	0
Decreased appetite	5 (17.9)	0	6 (30.0)	0
Constipation	8 (28.6)	0	2 (10.0)	0

* 1 TEAE in the SPd-40 group was missing grade.

SPd-40, 40 mg selinexor once weekly + 4 mg pomalidomide once daily + dexamethasone; SPd-60,60 mg selinexor once weekly + 4 mg pomalidomide once daily + dexamethasone; TEAE, treatment-emergent adverse event.

Nine patients (32.1%) in the SPd-40 cohort and 10 patients (50.0%) in the SPd-60 cohort had a dose reduction in selinexor after a median of 29.0 days (range 9-440) and 36.5 days (range 15-120). Dose delay or interruption in selinexor was reported in 17 patients (60.7%) in the SPd-40 cohort and 15 patients (75.0%) in the SPd-60 cohort after a median of 28.0 days (range 8-694) and 50.0 days (range 8-183). Nineteen patients (67.9%) in the SPd-40 cohort and 15 patients (75.0%) in the SPd-60 cohort had a dose modification in selinexor after a median of 23.0 days (range 8, 694) and 37.0 days (range 8, 183).

## Discussion

In recent years, the treatment landscape of RRMM is constantly evolving as newer treatment modalities and combinations are being developed and evaluated in clinical studies. However, at present, disease progression in RRMM is inevitable, as the probability for response is lower, and response duration shorter to each subsequent line of treatment (14, 15).

Antineoplastic drug dosage and treatment schedules are frequently reassessed with the accumulation of clinical trial data and real-world experience (11). Furthermore, adjustments to anti-cancer regimens, such as dose reductions, interruptions, or discontinuation of therapy are commonly implemented throughout the course of treatment, with the aim of sustaining an ongoing tumor response while concurrently enhancing tolerability to therapy and improving the patient’s quality of life.

Our analysis showed that both SPd-40 and SPd-60 showed efficacy in patients with RRMM, with a generally improved toxicity profile for SPd-40. Treatment response and CBR were numerically higher in the SPd-60 cohort, which could be due to the greater selinexor dosing, yet may also reflect the lower proportion of patients who were αCD38 mAb- and triple-class refractory. Despite these differences, PFS and OS were longer in the SPd-40 cohort with the median PFS of SPd-40 cohort doubled compared to the SPd-60 cohort. Of note, for SPd-40 both PFS (median 18.4 months) and OS (64% alive at 24 months) were longer than those reported by Brioli et al. for pomalidomide combinations post daratumumab, in which PFS and OS were 6 and 12 months, respectively (16), as well as for those reported by the MAMMOTH study for triple-class refractory RRMM (PFS: 3.4 months; OS: 9.3 months) (8) and the LocoMMotion study for triple-class exposed RRMM (PFS: 4.6 months; OS: 12.4 months) (9).

The lower weekly selinexor dosing (SPd-40) showed an improved AE profile, as well as a longer median duration of exposure to therapy and a higher median relative selinexor dose intensity (**Table 2**). Most non-hematologic TEAEs, including nausea, occurred less frequently in the SPd-40 cohort and were generally transient and reversible, suggesting better tolerance and risk-benefit profile at the lower 40 mg dose. This dose-response toxicity effect was also noted previously among the patients treated in the phase 3 BOSTON study, where patients who had a selinexor dose reduction experienced a higher median PFS compared to those who did not receive reduced dosage (16.6 versus 9.2 months) as well as general improvements in toxicities (17). These observations have ultimately informed the currently ongoing phase 3 study of SPd-40 compared to combined standard dosing of elotuzumab, pomalidomide, and dexamethasone in pomalidomide-naïve patients who were previously treated with 1 to 4 lines of anti-MM therapy and were exposed to an IMiD, a PI, and an anti-CD38 mAb (NCT05028348).

Pomalidomide, an oral IMiD agent, is approved in North America and Europe in combination with dexamethasone for the treatment of RRMM in adults who have received 2 or more prior lines of therapy, including both a PI and lenalidomide, and have confirmed PD after their last treatment. Pomalidomide in combination with dexamethasone, daratumumab, elotuzumab and isatuximab is approved for use in the third line or later (18, 19). In Europe, the combination of pomalidomide, bortezomib and dexamethasone is approved for adults with RRMM who have received one or more previous treatments including lenalidomide (19). Our analysis adds information on the effectiveness of triplet regimens based on pomalidomide and low-dose dexamethasone (Pd) in the evolving post-anti CD38 mAb treatment landscape, in which there is no standard of care (8, 9). Prior studies have reported an ORR of 28% and a median PFS of 3.7 months to Pd in patients with MM refractory to both bortezomib and lenalidomide (10). Our analysis has shown an ORR of 50% and a median PFS of 18.4 months for SPd-40 and an ORR of 65% and a median PFS of 9.5 months for SPd-60. Median OS was not reached in either cohort. These results compare favorably to a retrospective analysis of 30 patients with pomalidomide-naïve disease who were treated with pomalidomide-based combinations (most of them triplet combinations) after becoming refractory to daratumumab (the median number of previous treatment lines was 4), which reported an ORR of 53%, a median PFS of 6 months (95% CI 3.4, 8.5), and a median OS of 12 months (95% CI 3.3, 20.7) (16).

The limitations of the study include its non-randomized design as patients were assigned to different regimens to evaluate their safety and efficacy and each cohort had a different follow-up duration. The small number of patients must be taken into consideration when interpreting the results. There could also be potential differences due to population characteristics which is mitigated by the similar disease history inclusion criteria across the studies.

In conclusion, our analysis showed that the all-oral combination of weekly SPd in patients with RRMM was generally tolerable and suggests that the combination is effective in this patient population. This analysis, while not designed to demonstrate statistical superiority of SPd-40 vs SPd-60 in ORR, provides useful observations to guide selinexor dose in future clinical trials. Similar to the observations in the BOSTON study among the patients who had selinexor dose reductions (17), the SPd-40 group had a better AE profile versus the SPd-60 group. This improvement in tolerability may have led to numerically longer PFS and OS because it enabled more consistent dosing of selinexor. Observed ORR was greater in the SPd-60 cohort, but both PFS and duration of treatment were longer in the SPd-40 group despite a higher rate of triple-class refractory disease at baseline with the overall risk-benefit profile favoring the SPd-40 regimen.

## Data availability statement

The datasets presented in this article are not readily available because Karyopharm Therapeutics follows a procedure for evaluating and fulfilling requests for sharing company clinical trial data with qualified external scientific researchers to meet the company's obligation to protect the rights and privacy of trial participants consistent with the EFPIA/PhRMA Principles for Responsible Clinical Trial Data Sharing. Requests to access the datasets should be directed to medicalinformation@karyopharm.com.

## Ethics statement

The studies involving humans were approved by the institutional review board or an independent ethics committee at each participating center that participated in STOMP and XPORT-MM-028 trials. The studies were conducted in accordance with the local legislation and institutional requirements. The participants provided their written informed consent to participate in this study.

## Author contributions

DW: Data curation, Writing – review & editing. GS: Data curation, Writing – review & editing. SM: Data curation, Writing – review & editing. SL: Data curation, Writing – review & editing. EC: Data curation, Writing – review & editing. NL: Data curation, Writing – review & editing. DV: Writing – review & editing, Formal analysis. OB: Formal analysis, Writing – review & editing. MB: Writing – review & editing, Data curation.

## References

[1] Chari A Vogl DT Gavriatopoulou M Nooka AK Yee AJ Huff CA. Oral selinexor-dexamethasone for triple-class refractory multiple myeloma. N Engl J Med. (2019) 381:727–38. doi: 10.1056/NEJMoa1903455 31433920

[2] Grosicki S Simonova M Spicka I Pour L Kriachok I Gavriatopoulou M. Once-per-week selinexor, bortezomib, and dexamethasone versus twice-per-week bortezomib and dexamethasone in patients with multiple myeloma (Boston): A randomised, open-label, phase 3 trial. Lancet. (2020) 396:1563–73. doi: 10.1016/S0140-6736(20)32292-3 33189178

[3] Jagannath S Delimpasi S Grosicki S Van Domelen DR Bentur OS Špička I. Association of selinexor dose reductions with clinical outcomes in the boston study. Clin Lymphoma Myeloma Leuk. (2023) 23(12):917–23. doi: 10.1016/j.clml.2023.08.018 37743180

[4] Xpovio™ (Selinexor) Prescribing Information. Newton, MA: Karyopharm Therapeutics Inc. (2022).

[5] Bahlis NJ Sutherland H White D Sebag M Lentzsch S Kotb R. Selinexor plus low-dose bortezomib and dexamethasone for patients with relapsed or refractory multiple myeloma. Blood. (2018) 132:2546–54. doi: 10.1182/blood-2018-06-858852 PMC6302280 30352784

[6] Gasparetto C Schiller GJ Tuchman SA Callander NS Baljevic M Lentzsch S. Once weekly selinexor, carfilzomib and dexamethasone in carfilzomib non-refractory multiple myeloma patients. Br J Cancer. (2021) 126(5):718–25. doi: 10.1038/s41416-021-01608-2 PMC8605887 34802051

[7] White DJ Chen CI Baljevic M Tuchman SA Bahlis NJ Schiller GJ. Once weekly oral selinexor, pomalidomide, and dexamethasone in relapsed refractory multiple myeloma. Blood. (2021) 138:2748–. doi: 10.1182/blood-2021-148759

[8] Gandhi UH Cornell RF Lakshman A Gahvari ZJ McGehee E Jagosky MH. Outcomes of patients with multiple myeloma refractory to cd38-targeted monoclonal antibody therapy. Leukemia. (2019) 33:2266–75. doi: 10.1038/s41375-019-0435-7 PMC6820050 30858549

[9] Mateos M-V Weisel K De Stefano V Goldschmidt H Delforge M Mohty M. Locommotion: A prospective, non-interventional, multinational study of real-life current standards of care in patients with relapsed and/or refractory multiple myeloma. Leukemia. (2022) 36:1371–6. doi: 10.1038/s41375-022-01531-2 PMC9061296 35332278

[10] Miguel JS Weisel K Moreau P Lacy M Song K Delforge M. Pomalidomide plus low-dose dexamethasone versus high-dose dexamethasone alone for patients with relapsed and refractory multiple myeloma (Mm-003): A randomised, open-label, phase 3 trial. Lancet Oncol. (2013) 14:1055–66. doi: 10.1016/s1470-2045(13)70380-2 24007748

[11] Eaton KD Lyman GH. Dosing of Anticancer Agents in Adults. In: Hesketh JP Vora SR, editors. Uptodate. UpToDate Inc, Waltham, MA (2023).

[12] Kumar S Paiva B Anderson KC Durie B Landgren O Moreau P. International myeloma working group consensus criteria for response and minimal residual disease assessment in multiple myeloma. Lancet Oncol. (2016) 17:e328–e46. doi: 10.1016/S1470-2045(16)30206-6 27511158

[13] Rajkumar SV Harousseau JL Durie B Anderson KC Dimopoulos M Kyle R. Consensus recommendations for the uniform reporting of clinical trials: report of the international myeloma workshop consensus panel 1. Blood. (2011) 117:4691–5. doi: 10.1182/blood-2010-10-299487 PMC3710442 21292775

[14] Sonneveld P. Management of multiple myeloma in the relapsed/refractory patient. Hematol Am Soc Hematol Educ Program. (2017) 2017:508–17. doi: 10.1182/asheducation-2017.1.508 PMC6142583 29222299

[15] Yong K Delforge M Driessen C Fink L Flinois A Gonzalez-McQuire S. Multiple myeloma: patient outcomes in real-world practice. Br J Haematol. (2016) 175:252–64. doi: 10.1111/bjh.14213 PMC5096152 27411022

[16] Brioli A Gengenbach L Mancuso K Binder M Ernst T Heidel FH. Pomalidomide combinations are a safe and effective option after daratumumab failure. J Cancer Res Clin Oncol. (2023) 149:6569–74. doi: 10.1007/s00432-023-04637-x PMC10356885 36781500

[17] Syed YY. Selinexor-bortezomib-dexamethasone: A review in previously treated multiple myeloma. Target Oncol. (2023) 18:303–10. doi: 10.1007/s11523-022-00945-3 36622630

[18] Pomalyst® (Pomalidomide) Prescribing Information. Summit, NJ: Celgene, a Bristol-Myers Squibb company (2023).

[19] Imnovid® (Pomalidomide) Summary of Product Characteristics. Utrecht, Netherlands: Celgene (2023).

